# Assessment of the Diagnostic Accuracy of Limited CT Scan of Paranasal Sinuses in the Identification of Sinusitis

**DOI:** 10.5812/ircmj.1797

**Published:** 2012-11-15

**Authors:** Vahid Noorian, Arya Motaghi

**Affiliations:** 1 Department of General Surgery, Medical School, Shahid Beheshti University of Medical Sciences, Tehran, Iran; 2 Department of Radiology, Medical School, Shahid Beheshti University of Medical Sciences, Tehran, Iran

**Keywords:** Sinusitis, Spiral Computed Tomography, Diagnosis

## Abstract

**Background:**

Paranasal sinus CT has high sensitivity and specificity for sinusitis. However, this modality is costly and involves greater radiation exposure than plain radiographs.

**Objectives:**

We tried to compare 10-cut limited CT scan and standard CT scan in the diagnosis of sinusitis.

**Materials and Methods:**

We conducted a cross sectional case series from August to December 2010 on 150 patients with non-randomized sampling method in academic hospitals related to medical school of Shahid Beheshti University of medical sciences. Using standard CT scan as the gold standard, the sensitivity and specificity of limited series were calculated for each sinus group.

**Results:**

In our study limited CT scan had a sensitivity of 92%, specificity of 94%, positive predictive value of 90% and negative predictive value of 95%.

**Conclusions:**

The limited CT scan is useful for confirming the clinical diagnosis of sinusitis.

## 1. Background

Changes in the treatment of patients with sinusitis over the past 10 years have highlighted several aspects of imaging, which are important factors in the radiologic evaluation and diagnosis of sinusitis (1). To date, CT scanning has maintained its status as the imaging modality of choice for sinonasal inflammatory disease (1, 2). CT is the gold standard for exact delineation of inflammatory sinus disease and has become a routine radiological examination in the diagnosis of sinusitis. It presents complete information and an unparalleled sight of the sinuses, mainly the bony anatomy (2).

Furthermore, with improved technology modern multslice and helical CT scanners has become significantly faster, and the radiation dose has been considerably reduced (1). Paranasal sinus CT has high sensitivity and specificity for sinusitis. However, this modality is costly and involves greater radiation exposure than plain radiographs, and these features restrict its application (3-6). These limitations lead to modification of coronal CT scan, resulting in a variety of "limited” studies. Minimizing the level of radiation delivered to the patient is as important as obtaining a high-quality scan (3).

Radiologists have obtained methods to use lower mAs settings (mAs = milliamperes of current X scanning time) for CT, but generate the similar image quality, resulting in lower radiation exposure than occurs with normal settings. In addition, reducing the number of slices in routine contiguous paranasal CT is another approach that is used to diminish expenditure and radiation exposure. Various terms have been used such protocols including limited CT, screening coronal CT and CT mini-series (3). In our study from the complete sinus CT scan, the limited series were obtained by blocking from view all the other cuts and leaving the radiologist only 10 slice to read. The complete CT scan was the gold standard.

## 2. Objectives

The purpose of current survey was to determine the sensitivity, specificity, positive predictive value and negative predictive value of the Limited 10-cut coronal CT for the identification of sinusitis.

## 3. Materials and Methods

We conducted a cross sectional case series with non-randomized sampling method from August to December 2010 on 150 consecutive patients (79 women and 71 men. Age ranged between 20-67 years, mean age 40 years) who referred to the radiology departments of academic hospitals related to medical school of Shahid Beheshti University of medical sciences because of clinical suspicion of sinusitis. All patients were examined with helical CT scan of sinonasal cavities as the standard technique and the definite diagnosis was recognized by this method. The study was approved by the institutional review board.

**Figure 1:**
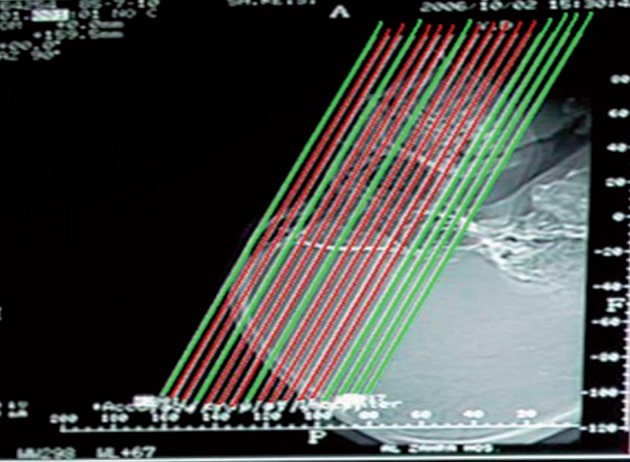
Standard CT protocol; coronal section with 2-3 mm collimation and 5 mm image interval. (Deleted slices is shown in green and limited series is marked as red)

The patients who could not hyperextend the neck and patients with history of functional endoscopic sinus surgery (FESS) were excluded. The helical CT images were obtained in coronal section with 2-3 mm collimation. Image interval was 5 mm through the ostiomeatal complex and further posterior sections (total 17 slices) ( Figure 1 ). The voltage was 120 kV, rotation time 3 S and tube current 60 mA.

The CT scan device was a third generation Toshiba-xvid, China. Each patient was scanned in the prone situation with his or her neck extended over the end of a table, and the scanning plane was set as perpendicularly to the hard palate as possible in order to achieve a true coronal plane. The radiological criteria for sinusitis were defined as opacification, air- fluid level, 3 mm or more of mucoperiosteal thickening, retention cysts, Ostiomeatal complex (OMC) patency, erosion and sclerosis of sinus walls. OMC included the frontoethmoidal recess, middle meatus, maxillary sinus infundibula, uncinate process and middle turbinate, which are the normal drainage of the paranasal sinuses.

**Figure 2:**
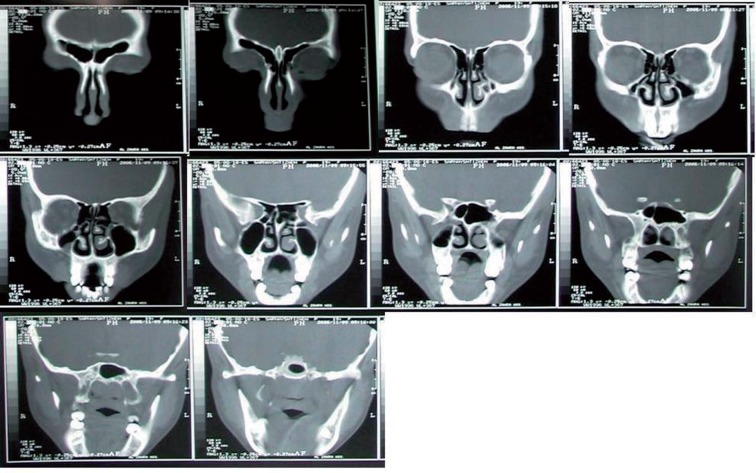
Selected coronal slices in the limited CT scan

Regarding to our standard protocol for paranasal sinuses CT scan, we deleted slices number 1, 4, 8, 14, 15, 16, 17 for limited series as indicated by judgment of our center’s practiced radiologists. Deleted slices were negligible in opposition to selected slices and were similar in all patients. The limited series were obtained by blocking from view all the other cuts and leaving the radiologist only 10 selected slices to read. Selected slices are shown in Figure 1 and 2. The CT hard copies were interpreted independently by two experienced radiologists. Divergent interpretations were discussed and final agreement achieved in all cases. Findings were recorded in a computer database, and by using standard CT scan as the gold standard, the sensitivity, specificity, positive predictive value (PPV) and negative predictive value (NPV) of limited series were calculated for the maxillary, frontal, group of ethmoid cells and compartment of sphenoid sinus. Genotype, distinguished by 4 bands (85, 97,108 and 205bp).

## 4. Results

The standard CT examination which is known as a gold standard was positive for sinusitis in 138 of 300 maxillary sinuses, in 60 of 300 frontal sinuses, in 54 of 300 sphenoid sinuses and in 120 of 300 ethmoidal cells. The corresponding numbers of true positive for limited series were 129, 48, 51 and 108 respectively. Limited CT scan was true negative on detecting sinusitis in 159 maxillary sinuses, in 231 frontal sinuses, in 237 sphenoid sinuses and in 165 ethmoidal cells ( Table 1 ).

**Table 1:** Comparisons of the standard and limited CT scans in the diagnosis of sinusitis in various sinuses

	Standard CT Scan	
	Abnormal	Normal
**Limited method in maxillary sinuses**		
Abnormal	129	3
Normal	9	159
**Limited method in frontal sinuses**		
Abnormal	48	9
Normal	12	231
**Limited method in ethmoidal sinuses**		
Abnormal	108	15
Normal	12	165
**Limited method in sphenoid sinuses**		
Abnormal	51	9
Normal	3	237

The sensitivity, specificity, positive predictive value (PPV) and negative predictive value (NPV) of the 10 cut limited CT examination for each sinus group are shown in Table 2. The overall sensitivity, specificity, PPV and NPV with regard to presence or absence of sinusitis for limited CT was 92, 94, 90 and 95% respectively.

**Table 2:** Sensitivity, specificity, PPV and NPV of limited CT scan in patients with sinusitis

Sinus Value	Maxillary sinus	Frontal sinus	Sphenoid sinus	Ethmoid sinus	Overall
**Sensitivity**	93%	80%	94%	90%	92%
**specificity**	98%	96%	96%	91%	94%
**Positive Predictive Value**	97%	84%	85%	87%	90%
**Negative Predictive value**	94%	95%	98%	93%	95%

## 5. Discussion

Diagnosing paranasal sinusitis is a frequent problem in general practice (1). Failure to identification of sinusitis can lead to significant complications such as cellulitis and osteomyelitis. The indication and the necessity for plain X-rays in diagnosis and further management of sinusitis has declined over the last decade (4, 7). Conventional X-ray findings are correlated with operative finding only in half of the patients with sinusitis (3). The limited sensitivity of these films has led to the increased use of paranasal sinus CT that presents detailed images of the sinuses and provides the examiner a clear view of the regions which are important in the pathogenesis of sinusitis. These regions, especially the anterior ethmoid cells and ostiomeatal complex, are not well delineated on conventional X- rays (3). Coronal CT with section thickness of 3 to 4 mm is the image modality of choice for sinusitis (8, 9). Computed tomography is regarded as a relatively high-dose diagnostic procedure. International efforts are underway to measure and reduce the radiation dose (5, 6). In the sinus regions, there is a high contrast difference between the structures of interest. This and the fact that sinusitis is not a condition where maximum image quality is critical for therapeutic decision making, allows attempts with low milliampere settings, thin sections and discontinuous scanning. Many studies have demonstrated that decreasing the number of CT slice results in lower cost and less radiation exposure (3). Authors have shown that limited CT techniques are comparable to contiguous paranasal CT for diagnosing rhinosinusitis but limited protocols have proven insufficient for surgical planning (2-4).

Goodman et al. developed a four- slice screening protocol with sensitivity and specificity of 93.3% and 89.3% respectively for detecting rhinosinusitis in any sinus (10). Awaida et al. used a similar four-slice protocol with sensitivity and specificity of 81 and 89% respectively, for detecting rhinosinusitis in any sinus (4). Cagici et al. used a three- slice protocol with sensitivity and specificity of 95.1 and 92.6% respectively. The sensitivity of three-slice CT in the frontal, ethmoid, maxillary and sphenoid sinuses was 100, 98.6, 94.1 and 97.8% respectively (3).

In this study, we tested a ten-slice limited CT by leaving the radiologist only 10 selected cuts to read and blocking from view all the other cuts. The sensitivity and specificity for diagnosing sinusitis in any sinus with our method were 92 and 94%. The sensitivity of the ten-slice CT in the frontal, ethmoid, maxillary and sphenoid sinuses was 80, 90, 93 and 94% respectively. Our findings are identical to Hagtvedt et al. (2) because of similar protocol and significantly higher than Awaida et al. (4) because of interpretation 6 more slices. Reducing the radiation dose of CT would enable more common usage of this technique for screening rhinosinusitis. Obtaining fewer slices per scan decreases the amount of the radiation dose given to the patient (1-3, 11). Multislice CT scan (MSCT) is applied progressively more worldwide,however, still numerous centers use third-generation systems for imaging the sinuses. MSCT with its low radiation dose in comparison with third-generation systems that used in present study can now be an alternative technique for limited CT series. Therefore, we recommend the usage of limited CT scan as an alternative when MSCT is not accessible.

It seems that in the non-operative management of sinusitis, obtaining fewer slice provide adequate data for detecting sinusitis. However, limited series should not be used instead of contiguous CT on patients undergoing endoscopic sinus surgery since knowledge of the anatomical details is essential prior to the surgical procedure in these groups of patients. Consequently, we conclude that limited series is helpful for confirming the clinical diagnosis of sinusitis. However, standard CT scan of paranasal sinus is still necessary for preoperative planning of endoscopic sinus surgery, evaluation of OMC anatomical details, clinically suspected of sinusitis in cases with normal limited scan and sinusitis complications.
